# From compost to culver: genome sequence and annotation of a cluster CQ1 *Gordonia* phage

**DOI:** 10.1128/mra.01066-23

**Published:** 2023-12-22

**Authors:** William K. Alexander, Rianna R. Allen, Jaden D. Anderson, Amber N. Brumfield, Tiffany M. Cook, Giana M. Dana, Gregory J. Ethridge, Emily C. Gailey, Rebekah A. Netzley, Joshua V. Nguyen, Phillip J. Souza, Briggs M. Yoder, Jamie R. Wallen, Maria D. Gainey, Tonya C. Bates, Ellen M. Wisner

**Affiliations:** 1 Department of Biological Sciences, University of North Carolina at Charlotte, Charlotte, North Carolina, USA; 2 Department of Chemistry and Physics, Western Carolina University, Cullowhee, North Carolina, USA; DOE Joint Genome Institute, Berkeley, California, USA

**Keywords:** bacteriophages, *Gordonia*, phage genome, actinobacteriophages

## Abstract

Phage Culver, with a siphovirus morphology, was isolated using *Gordonia terrae* CAG3. Culver is assigned to phage cluster CQ1 based on gene content similarity to actinobacteriophages. Notably, Culver is predicted to encode eight tRNAs, lysin A by two adjacent genes, and, unlike other CQ1 phages, two putative integrase genes.

## ANNOUNCEMENT

**Fig 1:**
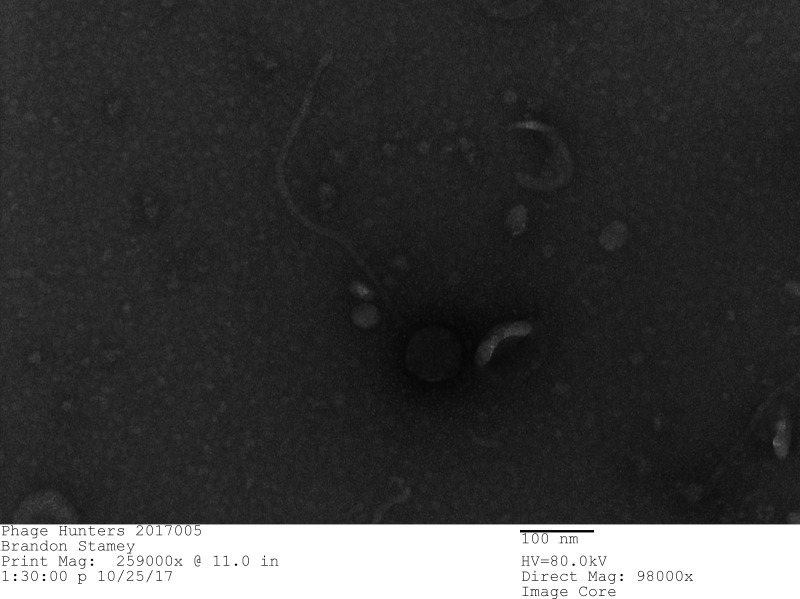
Bacteriophage particles were negatively stained with 1% uranyl acetate on 200 mesh carbon–formvar-coated copper grid and viewed with a FEI Tecnai BioTwin 120 keV TEM with digital imaging (Wake Forest University School of Medicine Cellular Imaging Shared Resource, Winston-Salem, NC, USA). Scale bar is 100 nm.

Understanding the immense diversity of bacteriophages provides valuable insights into the developing field of phage therapy used to combat antibacterial resistance (1, 2). Here, we present the characteristics of *Gordonia* phage Culver, adding to the understanding of actinobacteriophages (3). Culver was isolated from chicken coop compost soil in Cullowhee, NC (35.316700 N 83.164800 W) by students at Western Carolina University in 2017 using standard protocols (4). Briefly, the soil sample was suspended with peptone-yeast-calcium (PYCa) media and inoculated with *Gordonia terrae* CAG3. The sample was incubated with shaking for 48 h at 30°C, and then filtrate (0.22 uM) was plated with top agar and *Gordonia terrae*. Three rounds of plating (48 h incubations) were performed to purify Culver. Culver produced uniform, turbid plaques. Negative-stain transmission electron microscopy of Culver viral particles (Fig. 1) revealed a siphovirus morphology with a capsid 79.3 nm in diameter and a flexible long tail 450 nm in length (*n* = 1).

Genomic dsDNA was isolated from a lysate of Culver using a Promega Wizard DNA cleanup kit. The DNA was then prepared for sequencing using the NEB Ultra II Library Kit and later sequenced using an Illumina MiSeq sequencer (v3 reagents). This resulted in 234,297 150 bp length single-end reads, producing 344-fold coverage. Newbler v2.9 (5) and Consed v29 (6, 7) were used to assemble raw reads and finish the genome. Default parameters were used for all software. The genome length is 93,062 bp with defined 3′ single-stranded overhangs (CGCGACGCTC) as determined by Consed v29 and has a GC content of 61.9% (6, 7). Based on gene content similarity of at least 35% to phages in the Actinobacteriophage database (https://phagesdb.org/), using the phagesDB GCS tool and previously described criteria (8, 9), Culver was assigned to subcluster CQ1.

The Culver genome was auto-annotated using DNA Master v2705 (http://cobamide2.bio.pitt.edu) embedded with Genemark 2.5 p (10) and Glimmer 3.02 (11) and gene presence and start sites were manually refined using Starterator v509 (http://phages.wustl.edu/starterator/) and Phamerator v509 (12). Putative functions were assigned using NCBI BLASTp 2.13.0 with PhagesDB and NCBI nonredundant databases (13), DNA Master v2705, HHpred with PDB mmCIF20, UniProt SwissProt viral, and NCBI Conserved Domain (CD) v3.19 databases (14), DeepTMHMM v1.0.24 (15), SOSUI (16), and PECAAN 20210219 (discover.kbrinsgd.org). tRNAs were refined using Aragorn v1.2.41 (17) and tRNAscanSE v2.0 (18). Overall, 182 protein-coding genes were predicted and 55 were assigned a putative function. In addition, eight putative tRNAs were identified.

Interestingly, Culver has two putative tyrosine integrases (int-Y) (genes 60 and 85). Two integrases have been identified in a few other phages (19); however, all other currently annotated CQ phages only have one putative integrase gene. Similar to other cluster CQ phages, Culver’s predicted Lysin A is split into two adjacent genes, one containing the Lysin A protease C39 domain (gene 49) and the other containing the Lysin A L-Ala-D-Glu peptidase domain (gene 50).

## Data Availability

Culver is available at GenBank with Accession No. OR475265 and Sequence Read Archive (SRA) No. SRX19690869
